# Early Surgery: Le Fort I Advancement in a 6-Year-Old Patient—A Therapeutic Approach Using a Modified Le Fort I Osteotomy

**DOI:** 10.1007/s12663-023-01859-x

**Published:** 2023-02-14

**Authors:** Wolfgang Kater, Martin Trommlitz, Dorian Karnaus

**Affiliations:** 1 Clinic for Oral and Maxillofacial Surgery, Zeppelinstr. 24, 61352 Bad Homburg, Germany; 2 ENT practice at Specialist Center, Zeppelinstr. 24, 61352 Bad Homburg, Germany; 3 Justus-Liebig-Universität Giessen, JLU, Giessen, Germany

## Abstract

Orthognathic surgery in young patients before completion of skeletal growth is still sharply discussed today. In the following case report of a 6-year-old patient, however, there was a vital indication for treatment. The main clinical symptoms were characterized by impaired hearing as a result of constantly recurring seromucotympanum and adenoids, persistent rhinorrhea and otorrhea, chronic tonsillitis and chronic otitis media. ENT interventions such as the partial C-tonsillectomy, paracentesis with tympanic drainage, adenotomy and tube dilation with balloon catheter did not bring lasting success. Despite antibiotic therapy with aminopenicillins and cephalosporins in ß-hemolytic streptococci, no improvement in the symptoms could ultimately be achieved, so that there was a life-threatening risk of endocarditis with previous pulmonary valve replacement. In our orthognathic consultation, a maxillary retrognathism with a frontal crossbite was diagnosed. With an interdisciplinary consideration of the risks and side effects, an early surgical treatment in the sense of an upper jaw advancement with dilatation of the airways and evacuation of the maxillary sinuses was carried out. The operative challenge consisted of determining an ideal osteotomy line so as not to damage permanent tooth structures. Furthermore, the patient and his family had to understand the expected outcomes, potential risks, and possible complications that might arise from early surgical interventions, such as a subsequent maxillary growth discrepancy. After successful surgery the patient could already be discharged on the 2nd postoperative day and soon no longer showed any complaints or symptoms with regard to the tube ventilation disorder and the seromucotympanum—also no dental or skeletal recurrence has been evident up to now. With 25 years of experience in “Early surgery,” we have learned that orthognathic operations in children and adolescents might have decisive effects on life quality.

## Patient’s History

We report on a 6-year-old patient whose primary clinical symptoms were bilateral hearing impairment as a result of constantly recurrent seromucotympanum on both sides, recurrent adenoids, persistent rhinorrhea and otorrhea, hypertrophy of the conchae, chronic tonsillitis, rhonchopathy and chronic otitis media on both sides. Therefore, in the age of only 2 years and 4 months, a partial C-tonsillectomy, paracentesis and adenotomy had already been performed in November 2013 by an ENT Specialist. In September 2015, another paracentesis with adenotomy was necessary, but now with a tympanic drainage on the right.

In May 2016, the young patient underwent another operation due to the persistence and recurrence of the symptoms, this time radiofrequency ablation of the inferior nasal conchae (both sides), another paracentesis with T-tube insertion on both sides and tube dilatation with a balloon catheter. Despite antibiotic therapy using cephalosporins in laboratory-proven ß-hemolytic streptococci, no sustainable improvement in the symptoms could be recorded.

The patient suffered from a dysplastic pulmonary valve with severe stenosis, which had required a pulmonary valve replacement (RV-PA-Conduit / Contegra 20 mm) in June 2017.

The constant recurrence and chronification of the seromucotic tympanum, otitis media, adenoids, rhinorrhea and otorrhea have become problematic, since the risk of endocarditis due to the pulmonary valve replacement was significantly increased due to the permanent bacterial load. As a result, almost permanent antibiotic therapy using aminopenicillins and cephalosporins was carried out, which led to increasing multiple antibiotic resistances.

In March 2018, the patient presented in our jaw surgery consultation for the first time. Prima vista, a maxillary retrognathia, and the resultant mesial crossbite position of a premolar width (PB) could be diagnosed.

## Diagnosis

**Fig. 1:**
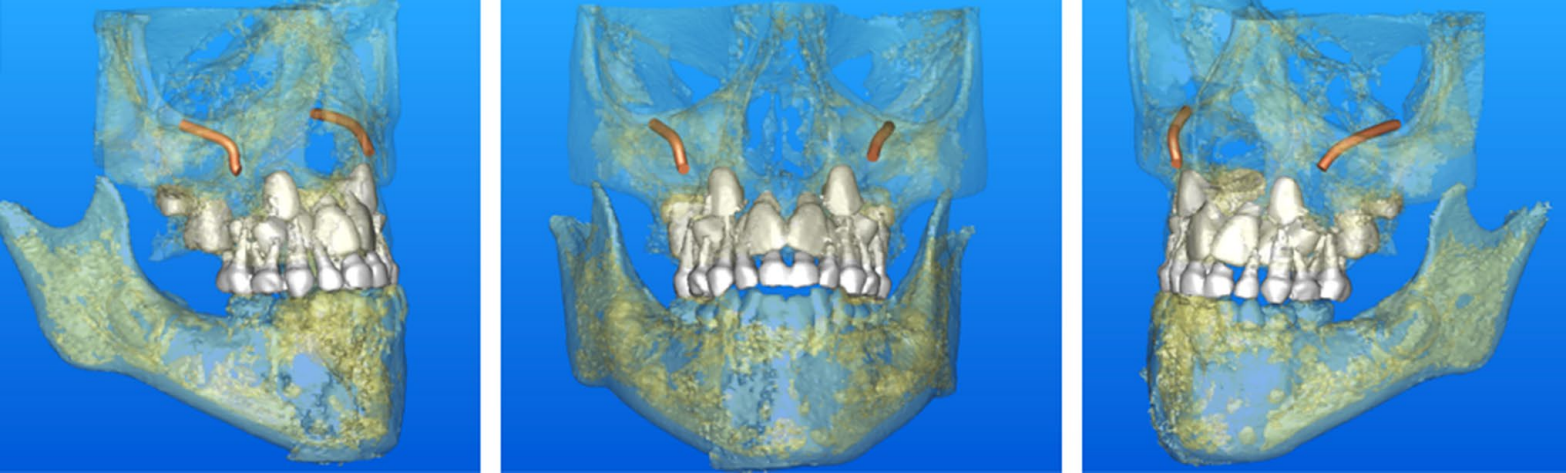
Preoperative 3D surface reconstruction of a CBCT showing the infraorbital nerves (orange), deciduous teeth and dental buds (white)

A performed digital volume tomography (Fig. 1) confirmed the diagnosis of maxillary retrognathism as the cause of the disruptive tube ventilation. The tooth buds of the 2nd dentition were developed according to age. Resistance to penicillin, amoxicillin and piperacillin has already been proved in the antibiogram.

## Treatment

With a team approach of the departments of orthodontics, radiology and ENT specialists, an interdisciplinary therapy concept was developed with a modified osteotomy to shift the midface in the Le Fort I plane in the sense of an upper jaw advancement. A purely orthodontic treatment without surgical intervention (Delaire’s mask [1, 2]) appeared to be less promising.

A combined orthodontic and maxillofacial surgical approach thus represented the last resort, since the patient's history of the dysplastic pulmonary valve and pulmonary valve replacement, as well as an acutely impending endocarditis, meant that a timely solution had to be found in order to treat the vitally endangered patient with a causal therapeutic approach.

In addition, there was a risk of endocarditis with multi-resistant pathogens.

With an interdisciplinary assessment of risks and side effects, early surgical treatment in the sense of maxillary advancement with rehabilitation of the airways and maxillary sinuses was therefore advocated.

Preoperatively, the model operation and simulation followed on April 26th, 2018, taking into account the age-appropriate tooth system of the permanent teeth that were still relatively far cranial. These were calculated and released using the Simplant® software (Dentsply Sirona; www.dentsplysirona.com/de-de/simplant.html). The ideal osteotomy line could now be determined in a modified, more cranially planned high Le Fort I osteotomy in order not to damage the permanent teeth. The cranial boundary was the infraorbital foramen with the infraorbital nerve on both sides, taking into account the anatomically difficult initial situation. The caudal boundary was defined by the dentition of the permanent teeth.

**Fig. 2:**
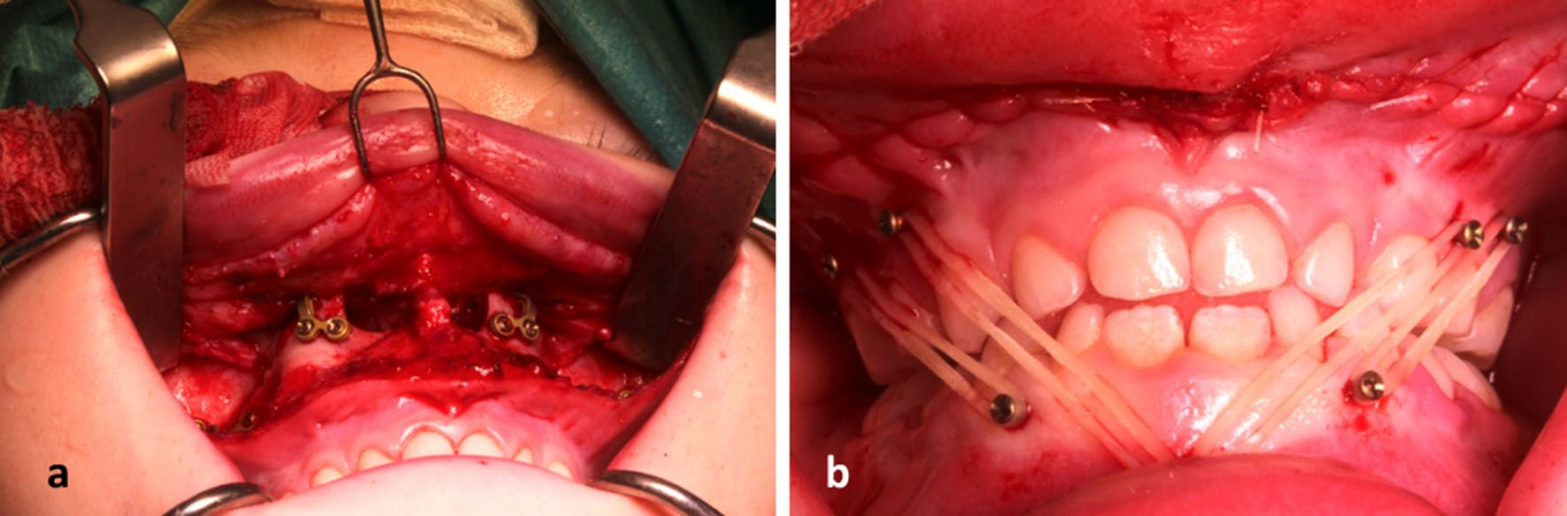
**a** Intraoperative picture showing micro-osteosynthesis plates fixed with microscrews on the zygomaticoalveolar crest and piriform aperture of the upper jaw. **b** Intraoperative picture showing the position of elastics on the self-tapping set TADs in class III position

Another challenge was the fixation using titanium microplates (Fig. 2a). To protect the three-dimensionally identified and localized permanent tooth system, we used self-tapping osteosynthesis microscrews.

The patient and his parents were informed in detail that a possible follow-up operation could be necessary after finishing of skeletal growth due to possible growth impairment.

The osteotomy to relocate the midface in the modified Le Fort I level as an upper jaw advancement with rehabilitation of the airways and maxillary sinuses carried out on May 4th, 2018.

The operation was performed under arterial hypotension and vasoconstriction (Xylonest 1% with added epinephrine (1:200,000)) in the entire surgical area. A modified Le Fort I osteotomy was performed with an oscillating saw under controlled arterial hypotension (RR < 80 mm Hg). Then, the upper jaw was separated from the sphenoid bone in the pterygopalatine fissures on both sides with the curved Obwegeser chisel, and the osteotomy of the nasal septum with the septum chisel. This was followed by the "down fracture" and mobilization of the upper jaw, which was typically performed very delicately and with extreme caution to avoid bleeding.

According to the planned dorsal impaction of the upper jaw, the dorsal, lateral and facial maxillary sinus walls as well as the lateral nasal walls were reduced, partly with the oscillating saw, partly with delicate bone punches. A particular difficulty here was the permanent tooth germs.

The nasal septum was now shortened and straightened, the spina nasalis was reduced and the nasal floor, which was severely constricted in the sense of a choanal stenosis, was widened with large round burs and placed deeper to compensate for the planned impaction. The bilateral inferior nasal conchae, which were hyperplastic, were resected caudally, and the mucosa located above them was closed with Vicryl 4/0 sutures.

Due to sinusitis, in both maxillary sinuses mucosa had to be removed. The surgical splint was fixed intermaxillary in the upper and lower jaw using transgingival titanium screws (TADs) (Fig. 2b). The titanium microplates are then aligned and fixed with 5 mm screws. The paranasal plates were doubled for better stability (Fig. 2a). Six titanium microplates (the paranasal plates were doubled) were required in the maxilla to ensure sufficient stability. The previously straightened nasal septum was fixed to the shortened spine via a drill hole caudal to the anterior nasal spine. The alae were attached to the nasal spine with Vicryl 2/0 sutures to narrow the alae spacing. In order to bridge the large bony gap and to avoid an ascending infection, Sulmycin implants were placed and fitted on both sides.

**Fig. 3:**
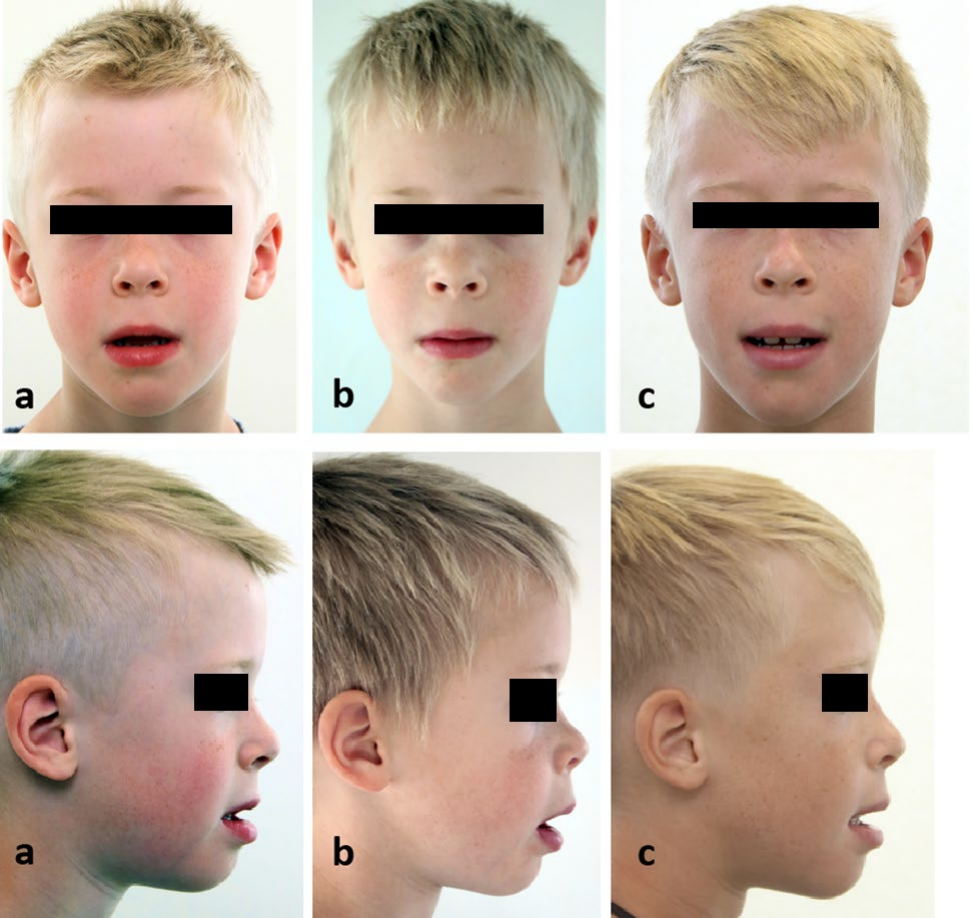
**a** Preoperative photodocumentation. **b** Photodocumentation 1 week after operation. **c** Follow-up photodocumentation 3 years and 1 month after operation

Swabs were taken from the right maxillary sinus, the left nostril, and the right auditory canal, from which copious amounts of pus emptied (Fig. 3).

Finally, because of the extreme tongue habit, an 11-mm mini-screw was inserted into the palate as a myofunctional stimulus.

## Postoperative

**Fig. 4:**
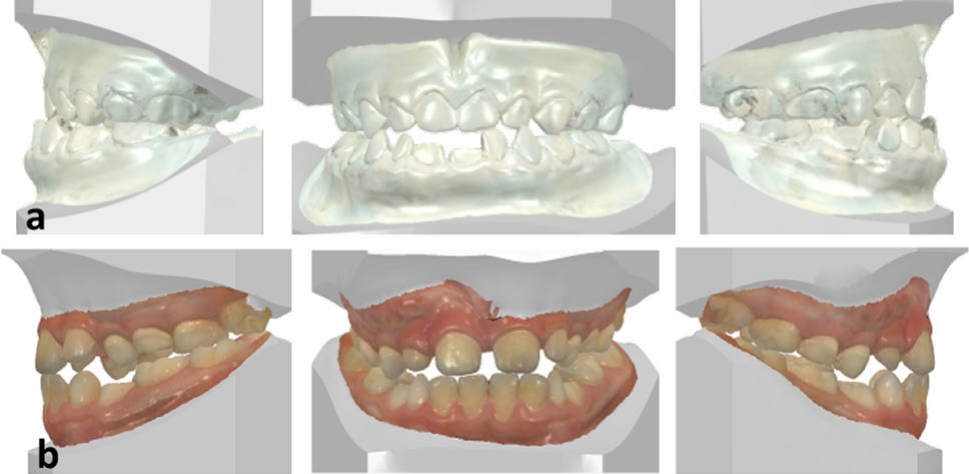
**a** Preoperative intraoral scan. **b** Follow-up intraoral scan 3 years and 1 month postoperative

The postoperative healing process with 2-day medication of 1 × 2 g ceftriaxone i.v.,  one-time medication of Solu-Decortin 50 mg as a decongestant  treatment and Hilotherm cooling device was uncomplicated. Furthermore, we did not use the surgical splint postoperatively. The patient could already be discharged on the 2nd postoperative day. The further clinical orthodontic and ENT follow-up checks showed an immediate improvement of the symptoms of the seromucotic tympanum, otitis media and tube ventilation disorder. Our patient was also able to confirm this subjectively, as he suddenly stated after the operation that he was finally able to hear much better, that he was more efficient in sports and at school and that he was sleeping undisturbed. Thus, the permanent antibiotic therapy could also be stopped (Fig. 4).

The anterior teeth 11 and 21 erupted 8 months postoperatively, the lateral anterior teeth 12 and 22 erupted 1 year and 5 months postoperatively in an age-appropriate manner and have properly settled in without damage and without delay.

## Discussion

There is much controversy regarding the timing of surgical correction of malocclusions. Many colleagues are reluctant to surgically correct developmental anomalies in the jaws before facial growth is complete. Waiting until skeletal growth is complete is justified for two reasons:

1. The surgical procedures required to correct the skeletal malformation may adversely affect subsequent growth [3]
2. Facial skeletal growth continues postoperatively, which could significantly affect the outcome of any surgery performed [4–7].

Conflicting results continue to be published, both advocating and discouraging an early surgical approach [8–14]. However, treating these patients with jaw abnormalities during their growth poses a challenging problem for both orthodontists and maxillofacial surgeons. One of the problems is the uncertain postoperative growth tendency, as there is no concrete consensus regarding the age limits for orthodontic or maxillofacial therapies [15]. The most compelling reason for early surgical correction before facial growth is finished is often the psychosocial component of the growing patient. Many children with severe jaw anomalies have problems being accepted by their peers, because facial appearance is an important factor in determining social relationships and affects the psychosocial perception of the child or adolescent [16, 17]. Therefore, early surgery during growth may be warranted and should be seriously considered to avoid negative psychological and/or psychosocial effects [16, 18, 19]. The potential benefits of early surgical correction of severe malocclusions also include a shorter treatment time, since no orthodontic phase treatment [20] is performed, and an increased healing potential [21]. Analysis of the growth rate and growth vector can be challenging, but it is necessary because the dysgrowth of the jaws often occurs in one but also in several dimensions. In general, female have completed about 98% of facial growth by the age of 15 and male by about the age of 17 [22, 23]. An understanding of facial growth tendencies and the specific anatomical face types (e.g., brachycephalic, normocephalic, dolichocephalic) provides important information about subsequent growth. Evaluation of the patient's medical and family history, as well as clinical and radiological examinations, is helpful in identifying growth disorders in the jaws [24].

Factors that can significantly affect the direction and rate of maxillary growth include genetics, developmental conditions, hormonal stimulants, and obstruction of the nasal or oropharyngeal airways [24–31]. The surgical management of the growing patient with maxillary anomalies continues to be the subject of much controversy.

The most common surgical procedure in the upper jaw to correct malocclusions is the Le Fort 1 osteotomy [24].

During this surgical procedure, the maxilla is separated from its bony cranial pillars (apertura piriformis, crista zygomaticoalveolaris, fissura pterygomaxillaris) and the nasal septum.

This surgical separation (referred to as a “down fracture” of the maxilla) effectively arrests further anteroposterior growth of the maxilla [32, 33].

Thus, if surgery is performed during the growing years, postoperative recurrence resulting in skeletal class III could occur if the mandible continues to grow normally.

If an early operation is nevertheless indicated for functional, esthetic and psychosocial reasons, a certain amount of overcorrection must be taken into account in the Le Fort I osteotomy of the maxilla so that the mandible, which is still growing, can develop and adjust properly in a natural way.

If this operation is performed during growth, the patients and their families must be fully informed that further operation is likely to be necessary at a later date [34]. Alternatively, the so-called "horseshoe osteotomy" (complete dentoalveolar osteotomy) of the upper jaw is under discussion. This osteotomy technique maintains the septal and vomeric connection in the maxilla because only the dentoalveolar mobilization is performed [24].

It is important to keep in mind that in patients who require maxillary advancement, there is insufficient maxillary growth preoperatively and there is no further anteroposterior growth, vertical maxillary growth, after the Le Fort I osteotomy, however, continues at the same preoperative rate postoperatively [33, 35, 36] and the mandible also continues to grow at the preoperative growth rate, which could again result in a Class III occlusal relationship [24].

However, severe functional or psychosocial factors may indicate earlier treatment. Both osteotomy procedures can technically be carried out in the first decade of life if there is sufficient space above the root tips of the developing permanent teeth or tooth germs to carry out the osteotomy and to carry out a sufficient osteosynthesis. Although vertical growth of the maxilla is unlikely to be affected by this procedure, damage to the developing tooth germs and roots can result in dentoosseous ankylosis and localized impairment of dentoalveolar growth [24].

### ENT aspects

The Eustachian tube protects against secretion, germ ascension and sound pressure from the nasopharynx, acts as a drain and serves to equalize pressure in both directions so that the eardrum and the sound conduction apparatus can vibrate optimally. Tubal dysfunction has an incidence of about 1% in adults and almost 40% in children. Symptoms are often nonspecific. In children, adenoid vegetations are often the cause of obstructive tubal dysfunction. In the case of the obstructive form, nasal sprays containing cortisone and the regular implementation of the Valsalva maneuver as well as tube dilatation with the Bielefeld balloon catheter are used therapeutically [37]. The typical symptoms of chronic obstructive tubal dysfunction are a feeling of pressure in the ears, aggravated by atmospheric pressure fluctuations, and difficulty in performing the Valsalva maneuver. Symptoms are often persistent and affected patients have long medical histories, which may begin in childhood. Long-lasting obstruction of the tube can lead to tympanic effusion and tympanic membrane retraction, often associated with a hearing loss (usually conductive hearing loss), and plays a crucial role in the pathogenesis of cholesteatoma [38]. Since patients with obstructive tube ventilation disorders tend to develop middle ear infections, they often suffer from the typical (late) consequences of recurrent or chronic inflammation of the middle ear [37]. Obstructive tube ventilation disorders are a common phenomenon in childhood. The predominant part is caused by adenoid vegetations. These constrict the torus tubarius and often sustain a local inflammatory reaction with mucosal swelling. In addition, the structure and the angle of inclination of the Eustachian tube are still different from that in adults up to about the age of 7: The cartilaginous part is larger, and the angle of ascent is flatter. The most important consequences of this tube dysfunction are serous to mucous tympanic effusions and recurrent otitis media. Favoring the development of cholesteatomas and adhesive processes is also discussed [37, 39]. The standard therapy consists of adenotomy and paracentesis, if necessary tympanic drainage. In the case of recurrent tympanic effusions and middle ear infections without recurrent adenoids or if the symptoms recur after tympanic drainage, balloon dilatation of the Eustachian tube can be considered as a second-line therapy [37, 39].

## Conclusion

**Fig. 5:**
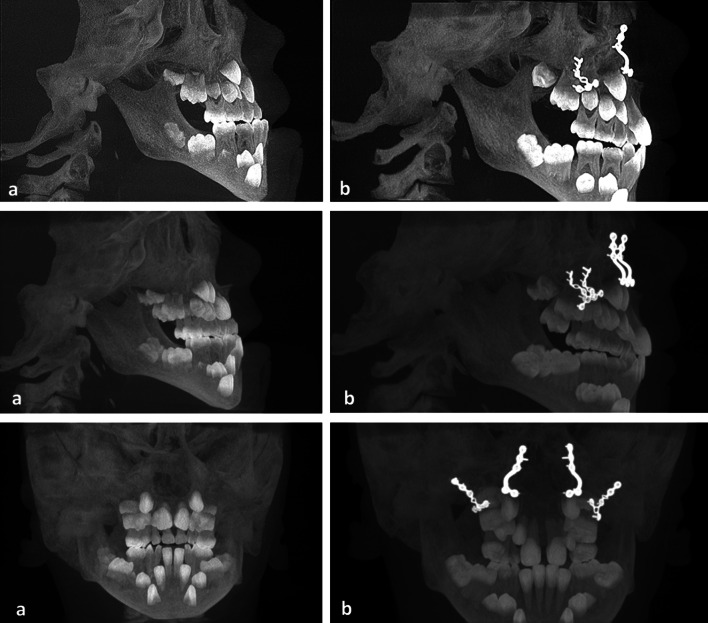
**a** X-ray images generated from a cbct scan 1 month and 7 days preoperatively. **b** X-ray images generated from a cbct scan 1 year and 5 months postoperatively

Treating growing patients with dentofacial abnormalities that require surgical correction presents orthodontists and surgeons with a unique and challenging problem. Children and adolescent patients with malocclusions may sometimes require surgical treatment during active growth due to functional, esthetic, and psychosocial factors, as well as vital treatment indications in this case. A well understanding of facial growth, available treatment options, and the impact of surgery on postoperative growth patterns when treating these patients is essential for good and desirable outcomes. Clinical, orthodontic model and X-ray analysis (Fig. 5a) are important in predicting individual patient growth rates and patterns. The type of the skeletal malformation present and the patient's specific growth vectors affect the surgical outcome and must be carefully evaluated prior to surgery. The patients and their families must understand the expected outcomes, potential risks, and potential complications that may arise from early surgical interventions. Factors such as the presence of mandibular disproportionate growth and co-existing temporomandibular joint disease can significantly affect postoperative growth and patient outcomes and must be identified and appropriately managed. In addition, facial growth can continue postoperatively and negate the outcome of any surgery that has been performed, leading to subsequent surgeries. This case report is in no way intended to be transferrable across the board, but rather to focus on alternative treatment methods in the case of such a diffuse medical history. It is particularly important to create a specific treatment plan for each young patient with regard to the appropriate type and timing of the corrective surgical intervention.

In summary, this case is a rarity due to the patient's age and the multifactorial interaction of antibiotic resistance, pulmonary valve replacement and tube ventilation disorders, which resulted in a life-threatening course.

However, since all known causal and surgical therapy options were carried out without lasting success and the general condition of the patient was increasingly deteriorating, also due to progressive antibiotic resistance, a rapid causal and vital therapy was required.

As a rule, it is not advisable to treat patients surgically before the eruption and placement of the permanent 12-year-molars in occlusion, with surgical treatment of skeletal malformation, since the risk of a “recurrence” is particularly high.

In this case, however, the osteotomy and displacement of the upper jaw to stretch and tighten the auditory tube and thus therapy of the seromucotympanum was the last resort as the only remaining vital treatment option.

Furthermore, our patient has no postoperative complaints or symptoms with regard to the tube ventilation disorder and the seromucotic tympanum. To date, no dental or skeletal recurrence has been identified.

The osteosynthesis plates were removed 1 year and 5 months after operation in order not to hinder the eruption of the tooth germs of the 2nd dentition.

Such orthognathic operations can in exceptional cases be carried out on younger patients, so the general attitude of waiting until skeletal growth is complete is no longer justifiable from today's point of view.

In summary, our long experience in severe cases encourages us to early surgery even in less severe cases as common procedure.

## Dedication

**Fig. 6:**
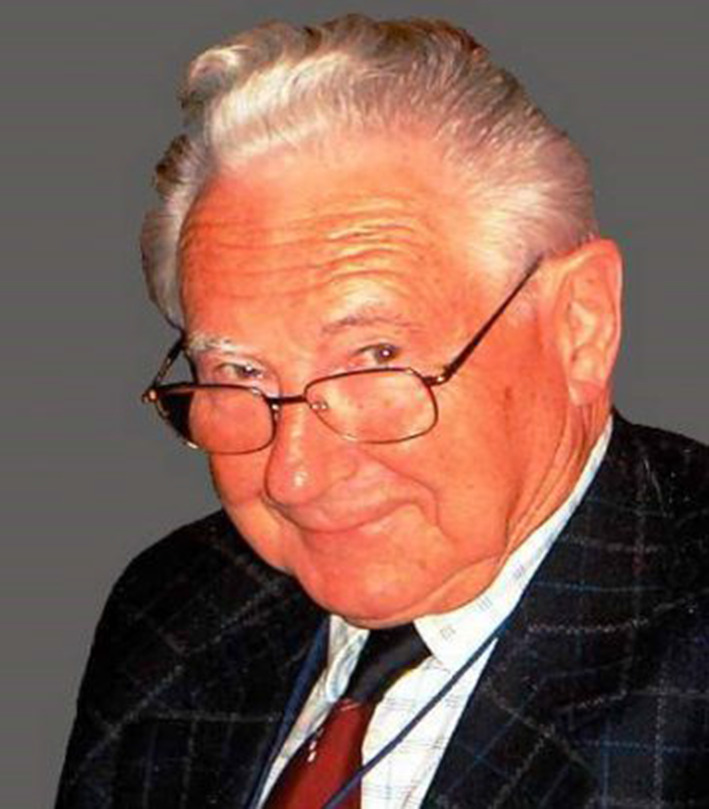
Prof Dr Jean Delaire (http://www.estmjs.org/members/jean-delaire/)

This paper is dedicated to Prof. Jean Delaire, who encouraged us in our efforts for early surgery (Fig. 6).

## References

[1] Amat P, Delaire J (2013). Traitement précoce des malocclusions de classe III: les convictions [Early treatment of class III malocclusions: conventional wisdom]. Orthod Fr..

[2] Delaire J (1988). L’emploi physiologique des tractions extraorales postéro-antérieures sur masque orthopédique dans le traitement des Classes III. Orthod Fr.

[3] McCarthy JG, La Trenta GS, Breitbart AS, Grayson BH, Bookstein FL (1990). The Le Fort III advancement osteotomy in the child under 7 years of age. Plast Reconstr Surg.

[4] Foley TF, Mamandras AH (1992). Facial growth in females 14 to 20 years of age. Am J Orthod Dentofacial Orthop.

[5] Love RJ, Murray JM, Mamandras AH (1990). Facial growth in males 16 to 20 years of age. Am J Orthod Dentofacial Orthop.

[6] Snow MD, Turvey TA, Walker D, Proffit WR (1991). Surgical mandibular advancement in adolescents: postsurgical growth related to stability. Int J Adult Orthodon Orthognath Surg.

[7] Stuzin JM, Baker TJ, Gordon HL (1992). The relationship of the superficial and deep facial fascias: relevance to rhytidectomy and aging. Plast Reconstr Surg.

[8] Epker BN, Fish LC, Paulus PJ (1978). The surgical-orthodontic correction of maxillary deficiency. Oral Surg Oral Med Oral Pathol.

[9] Epker BN, Wolford LM (1975). Middle-third facial osteotomies: their use in the correction of acquired and developmental dentofacial and craniofacial deformities. J Oral Surg.

[10] Freihofer HP (1977). Results of osteotomies of the facial skeleton in adolescence. J Maxillofac Surg.

[11] Kriens O (1974). Maxillary osteotomy in early childhood. A preliminary report. J Maxillofac Surg.

[12] Walker GF (1972). A new approach to the analysis of craniofacial morphology and growth. Am J Orthod.

[13] Wolford LM, Schendel SA, Epker BN (1979). Surgical-orthodontic correction of mandibular deficiency in growing children (long term treatment results). J Maxillofac Surg.

[14] Wolford LM, Walker G, Schendel S, Fish LC, Epker BN (1978). Mandibular deficiency syndrome. I. Clinical delineation and therapeutic significance. Oral Surg Oral Med Oral Pathol.

[15] Alwadei S (2017). Early orthognathic surgery: a review. J Contemp Dent Pract.

[16] Macgregor FC (1990). Facial disfigurement: problems and management of social interaction and implications for mental health. Aesthetic Plast Surg.

[17] Alley TR, Alley TR (1988). Physiognomy and social perception. Social and applied aspects of perceiving faces.

[18] Phillips C, Proffit WR, Proffit WR, White RP, Sarver DM (2003). Psychosocial aspects of dentofacial deformity and its treatment. Contemporary treatment of dentofacial deformity.

[19] Kiyak HA, McNeill RW, West RA (1985). The emotional impact of orthognathic surgery and conventional orthodontics. Am Orthod.

[20] Bishara SE, Ziaja RR (1989). Functional appliances: a review. Am J Orthod Dentofacial Orthop.

[21] Nanda R, Topazian RG, McNamara JA, Carlson DS, Ribbens KA (1982). Craniofacial growth following LeFort I osteotomy in adolescent monkey. Center for human growth and development.

[22] Broadbent BH, Broadbent BH, Golden WH (1975). Bolton standards of dentofacial developmental growth.

[23] van der Linden F (1986). Facial growth and facial orthopaedics.

[24] Mehra P, Wolford L (2016) Early orthognathic surgery: considerations for surgical management: principles, planning and practice. 10.1002/9781119004370.ch17

[25] Savara BS, Singh IJ (1968). Norms of size and annual increments of seven anatomical measures of maxillae in boys from three to sixteen years of age. Angle Orthod.

[26] Sillman JH (1964). Dimensional changes of the dental arches: Longitudinal study from birth to twenty-five years. Am J Orthod.

[27] Bjork A (1955). Facial growth in man studied with the aid of metallic implants. Acta Odont Scan.

[28] Scott JH (1963). The analysis of facial growth from fetal life to adulthood. Angle Orthod.

[29] Singh IJ, Savara BS (1968) Norms of size and annual increments of seven anatomical measures of maxillae in girls from three to seventeen years of age. Angle Orthod 312–24. 10.1043/0003-3219(1966)036<0312:NOSAAI>2.0.CO;2 5223796

[30] Bjork A, Skieller V (1972). Facial development and tooth eruptions: an implant study at the age of puberty. Am J Orthod.

[31] O’Reilly MT (1979). A longitudinal growth study: maxillary length at puberty in females. Angle Orthod.

[32] Freihofer HP (1977). Results of osteotomies of the facial skeleton in adolescence. J Maxillofac Surg.

[33] Mogavero FJ, Buschang PH, Wolford LM (1997). Orthognathic surgery effects on maxillary growth in patients with vertical maxillary excess. Am J Orthod Dentofac Orthop.

[34] Wolford LM, Karras SC, Mehra P (2001). Considerations for orthognathic surgery during growth. Part II: Maxillary deformities. Am J Orthod Dentofacial Orthop.

[35] Epker BN, Schendel SA, Washburn M, McNamara JA, Carlson DS, Ribbens KA (1982). Effects of early surgical superior repositioning of the maxilla on subsequent growth: III. Biomechanical considerations. The effect of surgical intervention on craniofacial growth.

[36] Washburn MC, Schendel SA, Epker BN (1982). Superior repositioning of the maxilla during growth. J Oral Maxillofac Surg.

[37] Schröder S, Ebmeyer J (2018). Diagnostik und Therapie von Funktionsstörungen der Tuba auditiva. HNO.

[38] Steinbach E, Pusalkar A, Heumann H (1988). Cholesteatoma pathology and treatment. Adv Otorhinolaryngol.

[39] Maier S, Tisch M, Maier H (2015) Balloon dilation of the Eustachian tube in pediatric chronic obstructive Eustachian tube dysfunction patients. HNO 63:686 (-688, S 690–684, S 696–687) 10.1007/s00106-015-0050-5 26311130

